# The pattern and treatment outcomes for rectal cancer with concurrent locoregional recurrence and distant metastases after total mesorectal excision

**DOI:** 10.1186/s12885-022-10212-3

**Published:** 2022-10-24

**Authors:** Yikuan Chen, Yaqi Li, Shaobo Mo, Xiang Hu, Fangqi Liu, Sanjun Cai, Xiaoji Ma, Junjie Peng

**Affiliations:** 1 Department of Colorectal Surgery, Fudan University Shanghai Cancer Center, 270 Dong’an Road, Xuhui, Shanghai, 200032 China; 2 Department of Oncology, Shanghai Medical College, Fudan University, Shanghai, 200032 China

**Keywords:** Locoregional recurrence, Distant metastasis, Rectal cancer, Treatment outcome, NED

## Abstract

**Background:**

To study the pattern and treatment outcome of rectal cancer (RC) with concurrent locoregional recurrence (LR) and distant metastasis (DM) after total mesorectal excision (TME) and to identify patient-, disease-, and treatment-related factors associated with differences in prognosis after concurrent LR and DM.

**Methods:**

RC patients who were diagnosed with concurrent LR and DM after TME from May 2015 to June 2019 were included in our study. All patients received single or multiple treatment modalities under the guidance of multidisciplinary team (MDT) of colorectal cancer in Fudan University Shanghai Cancer Center. The prognostic value of various clinicopathological factors for survival were calculated by Kaplan–Meier curves and Cox regression analyses.

**Results:**

A total of 74 RC patients with concurrent LR and DM who had undergone TME with a median follow-up of 27 months were eligible for analysis. The median survival of the included patients was 34 months, and 30 patients (41%) died. Fifty-nine patients (80%) underwent comprehensive treatments. Patients with oligometastatic disease (OMD) achieved no evidence of disease (NED) status more frequently than those with multiple metastases (*P* = 0.003). In the univariate analysis, patients achieving NED, diagnosed with OMD and five or less peritoneal metastases tended to have longer survival after LR and DM diagnosis (*P* < 0.05). In the multivariate analysis, attaining NED status was the only independent factor for survival (hazard ratio (HR), 2.419; *P* = 0.032). Survival after concurrent LR and DM in the non-NED group was significantly shorter than that in the NED group (median survival, 32 *vs.* 46 months; HR, 2.7; *P* = 0.014).

**Conclusions:**

The pattern and treatment outcome of RC with concurrent LR and DM after TME has changed with the development of multiple treatment modalities. Although the prognosis remains poor, pursuing NED status through comprehensive treatments may improve the survival of RC patients with concurrent LR and DM after TME.

## Background

Colorectal cancer (CRC) is the third most common cancer worldwide, and its overall 5-year survival rate is approximately 65% [1–3]. Approximately 50% of patients with CRC develop distant metastasis (DM) after curative resection, the most common of which is liver metastases [4, 5]. Rectal cancer (RC) accounts for 29% of all CRCs [2, 6]. Total mesorectal excision (TME) combined with pre-operative or postoperative radiotherapy (RT) or chemoradiotherapy (CRT) significantly reduces the locoregional recurrence (LR) rate in patients with RC to less than 10%, even 5% in some clinical centers [7, 8]. There are about 3% RC patients diagnosed with concurrent LR and DM after TME, which may cause severe disabling symptoms and usually have fatal outcomes [7–12].

For early and locally advanced RC, normative guidelines can be adopted for standardized treatment [7], while no consensus of treatment has been reached for concurrent LR and DM after TME. With the development of different treatment modalities, perioperative chemotherapy, palliative chemotherapy, targeted therapy, RT, radiofrequency ablation (RFA) and surgical resection can be applied singly or multiply [13–17]. However, several key problems have not been solved, including the sequence of local intervention and systemic treatment, the selection of surgical resection or RFA for local treatment, and the evaluation of tumors’ sensitivity to chemotherapy or RT. Thus, the individualized and comprehensive treatment of concurrent LR and DM still needs to be intensively studied.

Here, our study was designed to study the pattern and treatment outcome of RC with concurrent LR and DM after TME and to identify patient-, disease-, and treatment-related factors associated with differences in prognosis after concurrent LR and DM.

## Patients and methods

### Study design and patients

RC patients who were diagnosed with concurrent LR and DM after TME from May 2015 to June 2019 and fulfilled the following criteria were eligible for study entry: (i) aged from 18 to 80 years old at the time of diagnosis of concurrent LR and DM; (ii) diagnosed with resectable RC (histologically proven rectal adenocarcinoma) and received TME surgery with or without preoperative CRT; (iii) received treatment for LR and DM at Fudan University Shanghai Cancer Center (FUSCC); (iv) had complete medical records since RC diagnosis. After being diagnosed with concurrent LR and DM, all patients received single or multiple treatment modalities, including palliative chemotherapy, RT, RFA and surgical resection with or without preoperative chemotherapy under the guidance of multidisciplinary team (MDT) of CRC at FUSCC.

Patients were followed up regularly according to Chinese guidelines for CRC and ended at date of death or on December 31, 2019. Physical examination and carcinoembryonic antigen (CEA) were performed every 3–6 months for the first 2 years, every 6 months within the third to fifth year, and then annually. Chest/abdominal/pelvis computed tomography was performed annually for up to 5 years, and colonoscopy was performed for proper patients the first year after treatment and repeated in the third year if no advanced adenoma was found and then every 5 years. Clinical and pathological data were collected from electronic medical record system. Data on treatments and follow-up were gathered from surgeons, medical oncologists and radiologists. Patient data were collected prospectively using a standard form by researchers. All the follow up data of 74 patients are complete. The study was reviewed and approved by Institutional Ethics Committees of Fudan University Shanghai Cancer Center. All methods were carried out in accordance with relevant guidelines and regulations. Informed consent was obtained from all subjects and/or their legal guardian(s) before undergoing TME surgery and/ or treatment of recurrent disease. All the patients and/or legal guardians gave their consent that their data was used for this specific study.

### Evaluations of LR, DM and NED

LR was defined as radiologic and/or histologic evidence of a tumor within the lesser pelvis or the perineal wound after a macroscopically complete resection. LR location was categorized according to an adapted version of the subdivision proposed by Philipsen et al. [18] into recurrences located at the level of the anastomosis, regional lymph node and pelvic recurrences. DM was defined as radiologic and/or histologic evidence of a tumor in any other area. In this study, oligometastatic disease (OMD) was defined as DM in up to 2 organs or structures including liver, lung and localized lymph node, absence of ascites and peritoneal, bone and central nervous system metastasis. No evidence of disease (NED) status was defined as all LR and DM being grossly resected or ablated and with no sign of remnant disease at one month after surgery. Clear circumferential margin of local recurrence was not mandatory for determination of NED. Two fixed senior radiologists checked all images reported LR and DM.

### Statistical analyses

Chi-square tests were used to compare proportions, and Mann–Whitney U tests were used to compare continuous variables. Kaplan–Meier analyses were used to compare overall survival in patients between different groups. Cox regression was used for univariate and multivariate analyses with hazard ratios (HRs) and 95% confidence intervals (CI). Factors that were statistically significant in the univariate analysis were included in the multivariate analysis. *P* < 0.05 was considered as significant. Data on patients who were alive were censored at date of last contact. Because the aim of the study was to document the pattern and treatment outcome of RC patients with concurrent LR and DM after TME, the starting point for all survival analyses was the date of LR and DM diagnosis. All analyses were performed with SPSS statistical software (version 19.0 for Windows; SPSS Inc, Chicago, IL).

## Results

### Patients characteristics

**Fig. 1:**
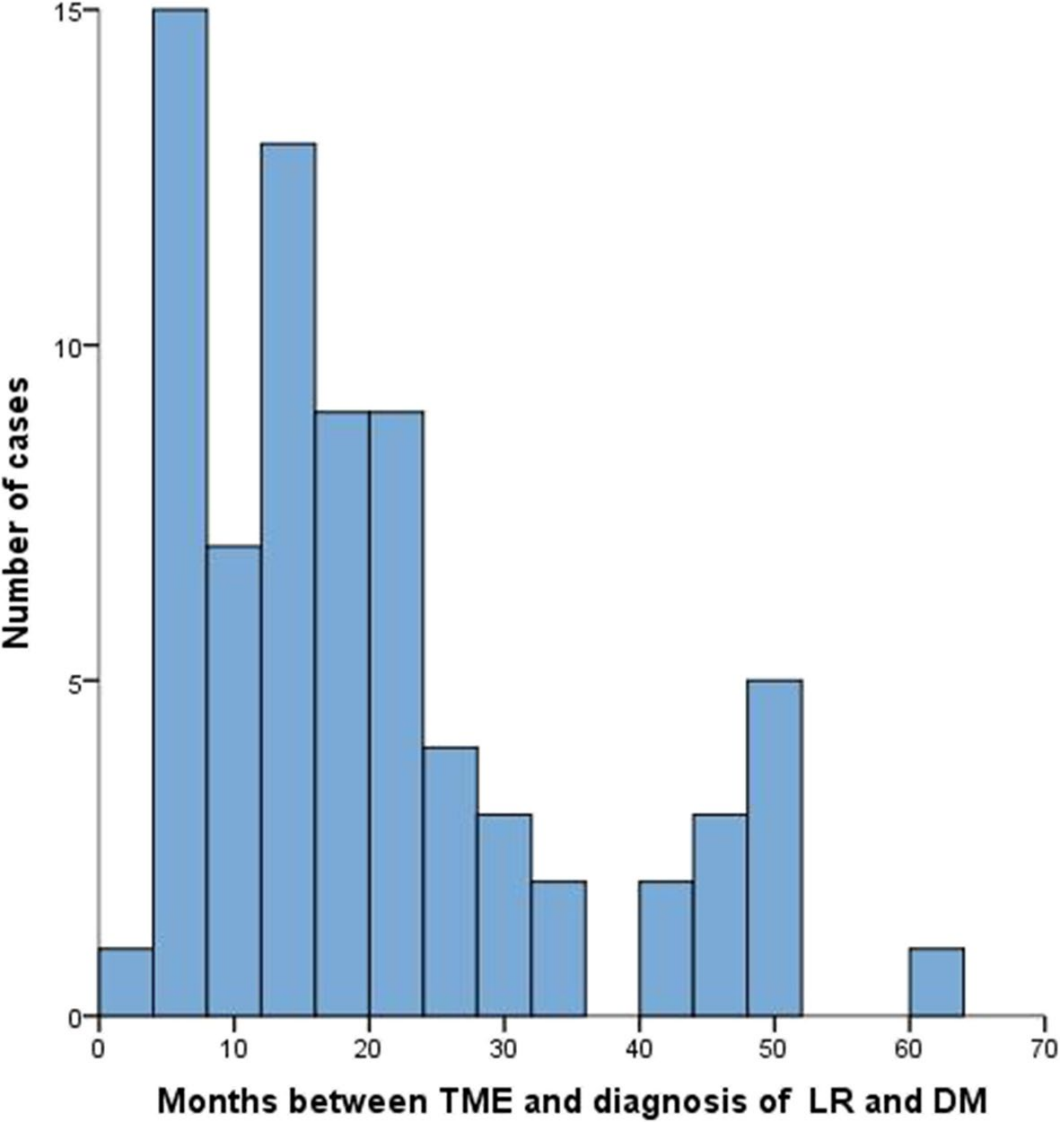
Time of diagnosis of concurrent locoregional recurrence (LR) and distant metastasis (DM) after total mesorectal excision (TME)

Among 8,376 patients with RC received TME surgery at FUSCC from May 2015 to June 2019, a total of 74 patients diagnosed with concurrent LR and DM were included in our study. The diagnostic rate of concurrent LR and DM in RC patients was 0.88%. Median time between date of LR and DM and date of primary tumor diagnosis was 16 months (range, 1 to 60 months) (Fig. 1).

The clinicopathological characteristics of 74 eligible patients were summarized in Table 1. Among all patients, half were aged over 60 years old. The primary tumor of 62.2% (46/74) patients were located over 5 cm from the anal verge. In terms of characteristics for the primary tumor, 91.9% (68/74) were diagnosed as T3-4, 60.8% as positive lymph nodes involved and 23.0% as G3 tumors. 74.3% (55/74) had anterior resection and 31.1% (23/74) had preoperative CRT or RT. For the type of local recurrence, 47.3% (35/74) of patients were diagnosed as regional lymph node recurrence while 31.1% (23/74) as anastomotic recurrence and 21.6% (16/74) as undetermined pelvic recurrence. For the type of distant metastasis, 71.6% (53/76) of patients were diagnosed with OMD and 28.4% (21/74) of patients were diagnosed to have metastases in 3 or more organs or in peritoneal. 73.0% and 85.1% of observed LR and DM had occurred within 2 and 3 years.

**Table 1:** Baseline characteristics of all eligible patients (*n* = 74)

Characteristics	No. (%)
Gender	
Male	45 (60.8)
Female	29 (39.2)
Age (years)	
< 60	37 (50.0)
≥ 60	37 (50.0)
Primary tumor location: distance from the anal verge (cm)	
> 5	46 (62.2)
≤ 5	28 (37.8)
Type of local recurrence	
Anastomotic recurrence	23 (31.1)
Regional lymph node recurrence	35 (47.3)
Undetermined pelvic recurrence	16 (21.6)
Distant metastasis	
Liver/lung/localized lymph node	53 (71.6)
3 or more organs/structures involved or peritoneal metastases	21 (28.4)
Type of resection of primary tumor	
Anterior resection	55 (74.3)
Abdominoperineal resection	13 (17.6)
Others	6 (8.1)
T stage of primary tumor	
T1-2	6 (8.1)
T3	41 (55.4)
T4	27 (36.5)
N stage of primary tumor	
N0	29 (39.2)
N1	36 (48.6)
N2	9 (12.2)
Tumor grade of primary tumor	
G1-2	57 (77.0)
G3	17 (23.0)
Preoperative CRT or RT of primary tumor	
Yes	23 (31.1)
No	51 (68.9)
Time to recurrence	
< 24 months	52 (70.3)
24–36 months	10 (13.5)
> 36 months	12 (16.2)

### Treatment modalities

Treatment modalities for 74 RC patients with concurrent LR and DM were listed in Table 2. 70 patients (94.6%) underwent at least one of the local treatments including surgical resection, RT and RFA. 48 patients (64.9%) received systemic treatments such as perioperative chemotherapy and palliative chemotherapy. 59 patients (79.7%) underwent multiple treatments. The results suggested that the vast majority of patients with LR and DM received comprehensive treatment no matter aggressively or palliatively.

**Table 2:** Treatment modalities of patients

Treatment modality										
Surgical resection	**√**	√	**√**	**√**	**√**					
Perioperative chemotherapy			**√**	**√**						
Palliative chemotherapy						**√**	**√**	**√**		**√**
Radiotherapy		**√**				**√**	**√**		**√**	
Radiofrequency ablation			**√**		**√**		**√**	**√**		
Cases	8	12	5	16	3	10	9	4	3	4
Percentage (%)	10.8	16.2	6.8	21.6	4.1	13.5	12.2	5.4	4.1	5.4

### Survival

**Fig. 2:**
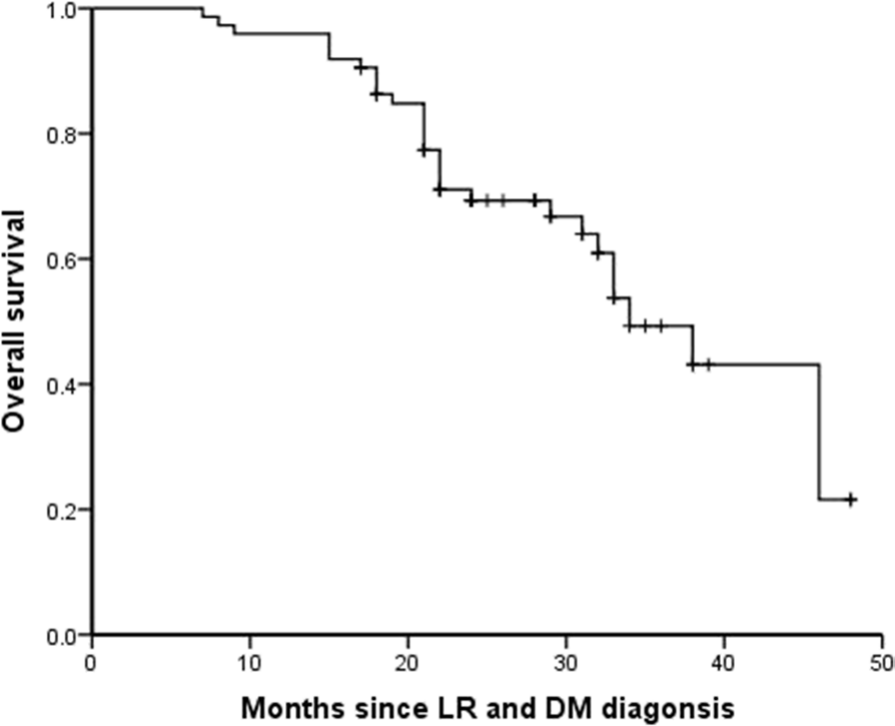
Overall survival of 74 patients after concurrent locoregional recurrence (LR) and distant metastasis (DM) diagnosis

During follow-up, 30 patients (40.5%) died and for the remaining 44 patients, median time between date of LR and DM diagnosis and date of last contact was 27 months (range, 17 to 48 months). Median survival after LR and DM diagnosis was 34 months (95% CI, 28.6 to 39.4 months) and three-year survival after LR and DM was estimated at 49.3% (Fig. 2).

**Fig. 3:**
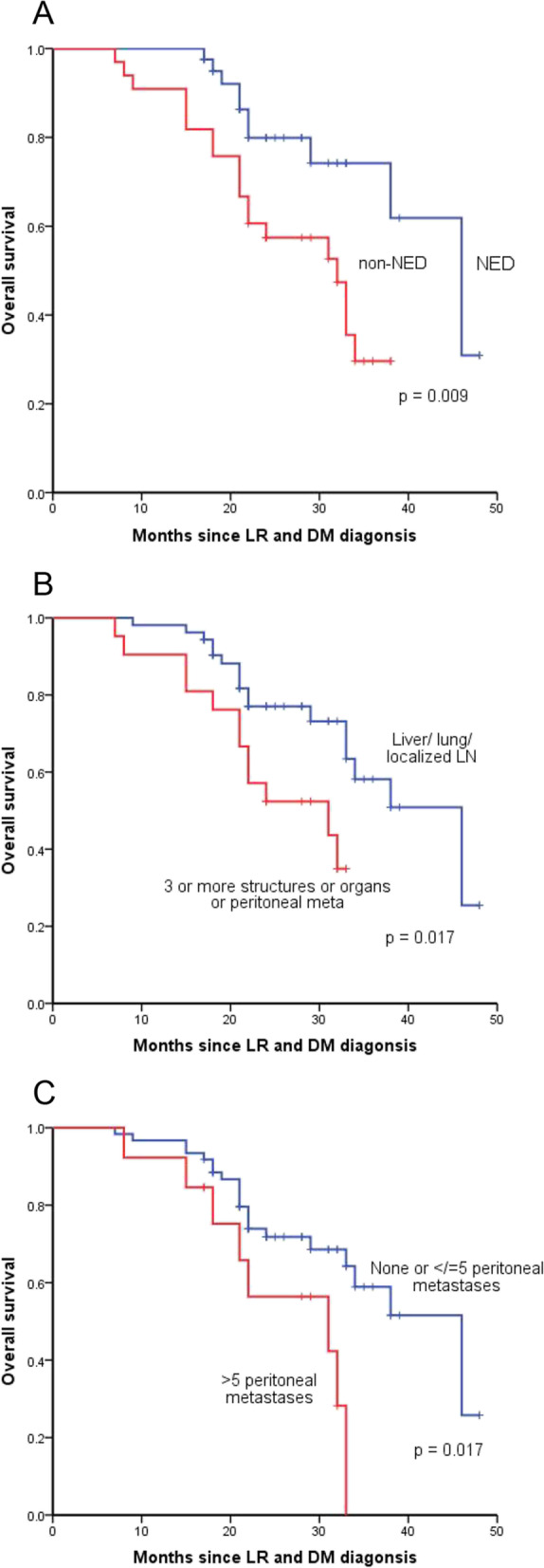
Kaplan–Meier analysis of overall survival in 74 patients after locoregional recurrence (LR) and distant metastasis (DM) according to no evidence of disease (NED) status (**A**), type of DM (**B**) and number of peritoneal metastases (**C**)

In univariate analysis, type of distant metastasis (HR, 2.464; 95%CI, 1.132–5.362; *P* = 0.023), number of peritoneal metastases (HR, 2.637; 95%CI, 1.140–2.229; *P* = 0.023) and NED status (HR, 2.727; 95%CI, 1.229–6.049; *P* = 0.014) were associated with survival (Table 3). Kaplan–Meier analysis showed that patients achieving NED (*P* = 0.009), diagnosed with OMD (*P* = 0.017) and five or less peritoneal metastases (*P* = 0.017) tended to have longer survival after LR and DM diagnosis (Fig. 3).

**Table 3:** Univariate and multivariate Cox regression analysis for survival

Related factors	Univariate			Multivariate		
	HR	95% CI	*P* value	HR	95% CI	*P* value
Gender			0.296			
Male	1.000					
Female	0.658	0.300–1.433				
Age (years)			0.333			
< 60	1.000					
≥ 60	0.691	0.327–1.460				
Primary tumor location: distance from the anal verge (cm)			0.856			
> 5	1.000					
≤ 5	0.933	0.443–1.965				
Type of local recurrence			0.159			
Anastomotic recurrence	1.000					
Regional lymph node metastasis	0.461	0.205–1.038				
Undetermined pelvic recurrence	0.565	0.182–1.751				
Distant metastasis			***0.023***			0.068
Liver/lung/localized lymph node	1.000			1.000		
3 or more organs/structures involved or peritoneal metastases	2.464	1.132–5.362		2.106	0.947–4.684	
Localized abdominal recurrence			0.060			
0	1.000					
1–3	0.695	0.279–1.734				
> 3	2.011	0.696–5.806				
Peritoneal metastases			***0.023***			0.513
0–5	1.000			1.000		
> 5	2.637	1.140–6.099		1.380	0.526–3.623	
Type of surgery of primary tumor			0.268			
Anterior resection	1.000					
Abdominoperineal resection	2.064	0.857–4.971				
Others	1.111	0.257–4.815				
T stage of primary tumor			0.362			
T1-2	1.000					
T3	1.668	0.220–12.624				
T4	0.965	0.121–7.668				
N stage of primary tumor			0.424			
N0	1.000					
N1	0.979	0.441–2.174				
N2	1.904	0.652–5.565				
Tumor grade of primary tumor			0.199			
G1-2	1.000					
G3	0.551	0.222–1.368				
Preoperative treatment of primary tumor			0.242			
Chemoradiotherapy or radiotherapy	1.000					
None	1.801	0.673–4.822				
Time to recurrence			0.233			
< 24 months	1.000					
24–36 months	3.074	0.835–11.315				
> 36 months	1.779	0.599–5.290				
No evidence of disease			***0.014***			***0.032***
NED	1.000			1.000		
Non-NED	2.727	1.229–6.049		2.419	1.078–5.427

After multiple variables adjustment in the Cox proportional hazards regression model, number of peritoneal metastases lost its statistically significance (HR, 1.380; 95%CI, 0.526–3.623; *P* = 0.513) and type of distant metastasis was marginal statistically significant for predicting survival (HR, 2.106; 95%CI, 0.947–4.684; *P* = 0.068) (Table 3). NED status was the only independent factor for survival after LR and DM diagnosis (HR, 2.419; 95%CI, 1.078–5.427; *P* = 0.032) (Table 3).

### NED status

The relationship between clinicopathological features and NED status was then analyzed (Table 4). The type of distant metastasis (*P* = 0.003), number of localized abdominal recurrence (*P* = 0.005), number of peritoneal metastases were all significantly related with NED status (*P* = 0.001). Thus, patients with OMD can achieve NED status more frequently.

**Table 4:** Association of NED status and clinicopathological features in 74 eligible patients

Characteristics	NED, n (%) (*n* = 41)	Non-NED, n (%) (*n* = 33)	*χ*^*2*^	*P* value
Gender				
Male	25 (33.8)	20 (27.0%)	0.001	0.974
Female	16 (21.6)	13 (17.6%)		
Age (years)				
< 60	17 (23.0)	20 (27.0%)	2.680	0.102
> / = 60	24 (32.4)	13 (17.6%)		
Primary tumor location: distance from the anal verge (cm)				
> 5	26 (35.1)	20 (27.0%)	0.061	0.804
≤ 5	15 (20.3)	13 (17.6%)		
Type of local recurrence				
Anastomotic recurrence	10 (13.5)	13 (17.6%)	2.115	0.347
Regional lymph node metastasis	22 (29.7)	13 (17.6%)		
Undetermined pelvic recurrence	9 (12.2)	7 (9.5%)		
Distant metastasis				
Liver/lung/localized lymph node	35 (47.3)	18 (24.3%)	8.525	***0.003***
3 or more organs/structures involved or peritoneal metastases	6 (8.1)	15 (20.3%)		
Localized abdominal recurrence				
None	10 (13.5)	4 (5.4%)	10.636	***0.005***
< / = 3	29 (39.2)	18 (24.3%)		
> 3	2 (2.7)	11 (14.9%)		
Peritoneal metastases				
None or < / = 5	39 (52.7)	22 (29.7%)	10.223	***0.001***
> 5	2 (2.7)	11 (14.9%)		
T stage of primary tumor				
T1-2	3 (4.1)	3 (4.1%)	1.161	0.560
T3	25 (33.8)	16 (21.6%)		
T4	13 (17.6)	14 (18.9%)		
N stage of primary tumor				
N0	17 (23.0)	12 (16.2%)	2.920	0.232
N1	17 (23.0)	19 (25.7%)		
N2	7 (9.5)	2 (2.7%)		
Tumor grade of primary tumor				
G1-2	30 (40.5)	27 (36.5%)	0.773	0.379
G3	11 (14.9)	6 (8.1%)		
Preoperative treatment of primary tumor				
Chemoradiotherapy or radiotherapy	16 (21.6)	7 (9.5%)	2.708	0.100
None	25 (33.8)	26 (35.1%)		
Treatment modality				
Single treatment	8 (10.8)	7 (9.5%)	0.033	0.857
Multiple treatment	33 (44.6)	26 (35.1%)		

Further survival analysis showed that 11 patients (26.8%) in NED group and 19 patients (57.6%) in non-NED group died during follow-up (Table 5). Three-year survival after LR and DM was estimated to be 61.8% in NED group and 29.6% in non-NED group. Patients in NED group have longer median survival after LR and DM diagnosis of 46 months (95% CI, 37.5 to 54.5 months), compared with that of 32 months (95% CI, 24.2 to 39.8 months) in non-NED group. Consequently, RC patients with concurrent LR and DM after TME have a poor prognosis, but reaching NED status after treatments can improve patients’ survival.

**Table 5:** Survival outcome of NED and non-NED group

Survival outcome	NED (*n* = 41)	Non-NED (*n* = 33)
Number of patients followed until death	11 (26.8%)	19 (57.6%)
3-year survival rate	61.8%	29.6%
Median survival time (month)	46	32

## Discussion

Though the incidence of concurrent LR and DM after TME of rectal cancer is quite low, which is 0.88% in our study, the prognosis of this subset of patients is poor. Our study retrospectively collected the pattern and treatment outcome of 74 RC patients with concurrent LR and DM after TME, to identify patient-, disease-, and treatment-related factors associated with differences in prognosis.

We found that the vast majority of patients with LR and DM received comprehensive treatment no matter aggressively or palliatively. Although the prognosis is still poor, pursuing NED status through comprehensive treatments may improve the survival of RC patients with concurrent LR and DM after TME. There are several possible explanations for this finding.

The first explanation concerns the treatment modalities. With development of multiple treatment modalities and MDT, the pattern and treatment outcome of RC with concurrent LR and DM after TME has changed. Our results indeed showed that the majority of patients (79.7%) underwent multiple treatments no matter aggressively or palliatively. Compared with Dutch trial in 2004 [9], more drugs with better clinical applications, more options for local and systematic treatment and modified therapy with LR and DM can be reached at present. For example, short-term preoperative RT (a total dose of 25 Gy in five fractions over 5 to 7 days) was used at that time, while long-term preoperative RT (a total dose of 45 Gy in 25 fractions over about 5 weeks) is widely used in FUSCC at present. Meanwhile, treatment of metastasis is more aggressive at present [19, 20]. With the development of treatment strategies, the median survival after LR and DM diagnosis was 34 months in our study and the median survival after LR 6.1 months in preoperative RT + TME group and 15.9 months in TME group in Dutch trial [9].

The second explanation concerns the survival. Although RC with concurrent LR and DM after TME has a poor prognosis, many studies have focused on attaining NED status after treatments to improve the overall survival which is also confirmed by our results. Furthermore, we found patients with OMD can achieve NED status after treatments more frequently. Consequently, patients with OMD after TME are the candidates to pursue NED status through upfront curative resection from the initial of the treatment, including CRT and RFA [21, 22].

The third explanation concerns surgical resection which is an important treatment modality to achieve NED status. However, if NED status not achieved, surgical resection of LR still plays an essential role. Due to the limited pelvic space, recurrent tumors are easy to compress other organs, such as ureter and blood vessels, leading to renal insufficiency and lower limb edema, which seriously affects the quality of life and subsequent treatment. Patients undergoing R0 resection have the greatest survival advantage following surgery for recurrent rectal cancer. Meanwhile, there is a survival advantage for R1 over R2 resection [23].

The present study has several limitations. First, the study design was a retrospective single-center trial. Second, this research does not include data for treatment intent. However, in the actual clinical treatment process, we made the choice of curative or palliative treatment intent upon initial diagnosis according to the LR and DM, whether it was resectable, whether it was OMD, the patient’s physical condition, the patient’s own will, and other factors, combined with MDT discussion opinions. Third, we defined OMD as metastasis in up to 2 organs or structures including liver, lung and localized lymph node, without taking the number, size of tumors into account. In ASCO-GI 2020, OMD was defined as up to 5 metastasis, up to 3 metastasis in one organ, up to 3 affected organs, size ≤ 3 cm, absence of ascites and peritoneal, bone and central nervous system metastasis [24]. Thus, it is possible that less patients were counted into OMD status.

## Conclusions

In conclusion, our study showed that RC patients with concurrent LR and DM after TME have a poor prognosis. Patients with OMD are the candidates to pursue NED status through multiple treatments including curative resection which may improve the overall survival.

## Data Availability

The datasets used and/or analysed during the current study available from the corresponding author on reasonable request.

## Abbreviations

CRC: Colorectal cancer
RC: Rectal cancer
LR: Locoregional recurrence
DM: Distant metastasis
TME: Total mesorectal excision
MDT: Multidisciplinary team
OMD: Oligometastatic disease
NED: No evidence of disease
HR: Hazard ratio
RT: Radiotherapy
CRT: Chemoradiotherapy
RFA: Radiofrequency ablation
FUSCC: Fudan University Shanghai Cancer Center
CEA: Carcinoembryonic antigen
CI: Confidence interval
